# Water Absorption and Hygrothermal Aging Behavior of Wood-Polypropylene Composites

**DOI:** 10.3390/polym12040782

**Published:** 2020-04-02

**Authors:** Wei Wang, Xiaomin Guo, Defang Zhao, Liu Liu, Ruiyun Zhang, Jianyong Yu

**Affiliations:** 1 Key Laboratory of Textile Science Technology, Ministry of Education, College of Textiles, Donghua University, Shanghai 201620, China; 1152020@mail.dhu.edu.cn; 2 Shandong Academy of Sciences Institute of Information, Qilu University of Technology (Shandong Academy of Sciences), Jinan 250014, China; 3 College of Textile and Garment, Shaoxing University, Shaoxing 312000, China; 4 Innovation Center for Textile Science and Technology, Donghua University, Shanghai 201620, China

**Keywords:** wood powder, polypropylene, water absorption, degradation, hydrothermal aging

## Abstract

Environmentally sound composites reinforced with natural fibers or particles interest many researchers and engineers due to their great potential to substitute the traditional composites reinforced with glass fibers. However, the sensitivity of natural fiber-reinforced composites to water has limited their applications. In this paper, wood powder-reinforced polypropylene composites (WPCs) with various wood content were prepared and subjected to water absorption tests to study the water absorption procedure and the effect of water absorbed in the specimens on the mechanical properties. Water soaking tests were carried out by immersion of composite specimens in a container of distilled water maintained at three different temperatures, 23, 60 and 80 °C. The results showed that the moisture absorption content was related to wood powder percentage and they had a positive relationship. The transfer process of water molecules in the sample was found to follow the Fickian model and the diffusion constant increased with elevated water temperature. In addition, tensile and bending tests of both dry and wet composite samples were conducted and the results indicated that water absorbed in composite specimens degraded their mechanical properties. The tensile strength and modulus of the composites reinforced with 15, 30, 45 wt % wood powder decreased by 5.79%, 17.2%, 32.06% and 25.31%, 33.6%, 47.3% respectively, compared with their corresponding dry specimens. The flexural strength and modulus of the composite samples exhibited a similar result. Furthermore, dynamic mechanical analysis (DMA) also confirmed that the detrimental effect of water molecules on the composite specimens.

## 1. Introduction

Bio-sourced materials, the most abundant resources on the planet, are emerging as reinforcing materials for composites, which have attracted much scientific and industrial attention owing to their attractive advantages such as renewability, biodegradability, sustainability, low density, low cost and better mechanical properties [1,2,3,4,5]. At present, the most common reinforcing material is glass fibers, but the manufacturing process of glass fibers and their products is harmful to the human body, such as dermal and respiratory irritation, and even cancer [6,7,8]. Moreover, glass fibers are non-renewable and non-biodegradable, with a density approximately twice that of natural plant fibers or particles. Therefore, natural plant materials have great potential to replace glass fibers as composite reinforcements.

However, one of the main disadvantages of natural fiber reinforced thermoplastic composites is the poor interfacial compatibility between natural fiber materials and polymers [9,10,11]. Taking wood powder reinforced polypropylene composite materials as an example, the wood powder has lots of hydroxyl groups, which makes the wood powder easy to combine with water molecules in the surrounding environment and so the wood powder exhibits excellent water absorption. However, polypropylene, as a thermoplastic, has no polar groups. In this way, the compatibility of hydrophilic wood powder and hydrophobic polypropylene is poor at the interface. Therefore, some appropriate modification methods of the surface of fibers or matrices need to be used to enhance the interfacial bonding [12,13,14].

Wood powder is very rich in sources and simple in processing, which mainly contains lignin and wood fibers. Wood fibers have high strength and elasticity, while lignin has good strength and rigidity, which make wood powder very suitable as a filling material for polymers. At the same time, the use of wood powder provides a method for the treatment of waste wood like waste furniture, because waste wood can be used to process wood powder. In this way, the secondary utilization of wood resources can be realized, and environmental problems can be avoided. Another important component, polypropylene (PP), as a kind of common engineering thermoplastic, can be recycled after service time, for example, PP fibers used in textile or other industries can be recycled into granulation and reused in polymer matrix composite materials. However, the strength and modulus of polypropylene are relatively low. Therefore, injection molding or other molding methods can be used to fill the wood powder into the polypropylene to exert the reinforcing effect of wood powder on the polypropylene. On the other hand, the polypropylene can also wrap the wood powder and bond them together. Even more importantly, the PP-based composites filled with wood powder can also be recycled after service time, which is superior to traditional high-performance or glass fiber composite materials in this respect.

Whether it is wood powder or polypropylene, they absorb moisture when in a humid environment or when immersed in water [7]. Unfortunately, moisture uptake will cause the material to degrade, especially in the interface of composites. The damage of the interface of composites will affect the stress transfer, resulting in reduced mechanical properties of the composite. Therefore, the study of the moisture uptake behavior of natural fiber-reinforced composites and the effect of moisture on the mechanical properties is very important.

It is generally believed that there are three different mechanisms for the diffusion of moisture in polymeric composites, namely, the diffusion of water molecules in the micro gaps between the polymer chain, the capillary transport of water molecules in the interface between the fiber and the polymer, and the transport of water molecules in the defect or micro gaps of the polymer [7,15,16]. The diffusion of water molecules is the main process in most cases though the three mechanisms take effect jointly. Previous researches on the water absorption of natural fiber-reinforced composites have shown that it can be divided into three cases according to the relative motion rate of water molecules diffusion and polymer segments, namely Case I (Fickian diffusion, in which the diffusion rate of water molecules is much less than that of the polymer segment mobility), Case II (and Super case II, in which the diffusion rate of water molecules is much larger than that of the polymer segments) and non-Fickian (anomalous diffusion, where the diffusion rate of water molecules is comparable to that of the polymer segments) [15,16,17,18,19,20].

A lot of studies on the water absorption of natural fiber-reinforced polymer composites have been carried out. The degradation effect of water molecules on the composites has been confirmed and the mechanical properties of the composites are affected by the water absorbed [21,22,23,24,25]. It is known that the mechanical properties of the natural fiber-reinforced polymer composites can be improved by the modification of fibers or polymers [26,27]. What is more, several efforts have shown that the modification of the interface between natural fibers and polymers can also reduce the degradation effect of moisture on some mechanical properties [15,28].

However, many of them focused on the short fiber reinforced polymer composites such as flax, sisal and hemp. In this paper, the water uptake behavior of wood powder filled polypropylene composites and the effect of water uptake on the mechanical properties were studied. The tensile and flexural tests were employed to characterize the change of the mechanical properties. More importantly, the dynamical mechanical analysis (DMA) was used to characterize the interface of the composites and study how the water molecules degraded the mechanical properties of the composites.

## 2. Materials and Methods

### 2.1. Material Preparation

Polypropylene (PP) pellets (Melt Flow Rate at 230 °C/2.16 kg of 3 g/10 min and density of 0.9 g/cm^3^) were used for injection molding, which supplied by Prime Polymer Co., Ltd., Osaka, Japan. And the compound pellets of wood powder and PP were employed, which contained 70 wt % wood powder and can supply wood power in the experiment. Moreover, Maleic anhydride grafted polypropylene (MAPP) was used to improve the interfacial properties of wood powder and PP.

Samples used for investigating the water absorption behavior of wood powder-filled polypropylene composites were prepared by injection molding. All the raw materials used for injection were dried in the desiccator to remove the moisture inside the materials until the weight reached the balance. Then the pellets were mixed evenly and then they were fed into an injection molding machine (TOYO MACHINERY & METAL CO., Ltd., TI-30F6, Akashi, Japan) with different mass ratios to obtain the composites including corresponding wood content. The specific process parameters were as follows: the rotation speed and the forward speed of the screw were 185 rpm and 30 mm/s respectively, the barrel temperature was 160–170 °C, and the molding temperature was 30 °C.

**Figure 1:**
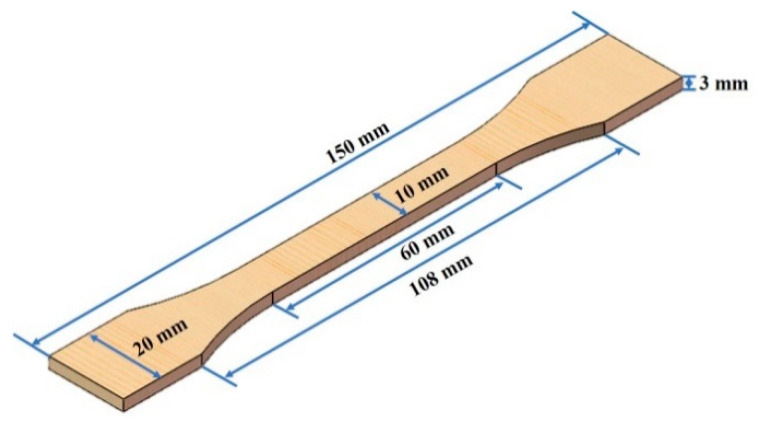
Dimensions of the test samples.

The composite samples with 0%, 15%, 30% and 45% by weight wood powder content were prepared, which were dumbbell-shaped (the nominal thickness and width were 3 and 10 mm, respectively), and the specification of the composites is shown in Figure 1.

### 2.2. Water Uptake Tests

Natural fiber composite materials are relatively sensitive to moisture, so understanding the degree of water absorption and the process of water absorption of natural fiber composite materials is of great significance for its application. The water absorption behavior is related to temperature, so different temperatures were employed in this study. The water uptake experiments were carried out in accordance with the ASTM D 570 standard. First, five specimens for each group sample used in the water uptake test were dried at 80 °C for 24 h. Then, the specimens with 0%, 15%, 30% and 45% by weight wood powder content were placed in four identical containers respectively and immersed in distilled water at a temperature of 60 °C. The specimens with 45% by weight wood powder content were selected to study the effect of temperature on the water absorption behavior of the composite samples, therefore, they were also immersed in distilled water at 23 and 80 °C, respectively. The specimens were taken out from water after a period, and they were weighed in a high precision balance (accuracy of 0.1 mg) after removing off the water on the surface with an absorbent lint-free cloth. After that, the specimens were immersed in distilled water again. The weight gain (water content) was obtained by the weight difference. Repeating this until the sample mass was almost constant.

### 2.3. Mechanical Characterization of Samples

The tensile tests of both dry and wet specimens were performed using an electronic universal testing machine (AGS-X, Shimadzu Corp., Kyoto, Japan) equipped with a 1 kN load cell. The span length was set as 115 mm, the crosshead speed was set as 2 mm/min, and the whole tests were in a standard laboratory (the temperature of 23 °C and the relative humidity of 50%). Five specimens for each configuration were measured and the average values were reported.

The flexural tests of both dry and wet specimens were conducted using the three-point bending mode with a universal testing machine (Instron, Norwood, MA, USA). The support span and the crosshead speed were 48mm and 2mm/min respectively. The testing environmental conditions are the same as the tensile tests. Five specimens were tested and the average values were recorded.

### 2.4. Dynamic Mechanical Analysis (DMA) of Samples

The dynamic thermal mechanical behavior of both dry and wet samples with the required dimensions (length, width and thickness of 60 mm, 10 mm and 3 mm respectively), which cut from the injection-molded specimens was measured by DMA 2980 (TA Instruments, New Castle, DE, USA). The tests were employed in a double cantilever mode with a heating rate of 3 °C/min from −60 to 80 °C, frequency of 1 Hz, and amplitude of 15 μm.

## 3. Results and Discussion

### 3.1. Water Uptake Behavior

The water content of the specimens can be calculated by the weight difference between samples before and after water uptake, which can be expressed by the equation as follows:

(1) $$W_{w}=\frac{W_{t}-W_{d}}{W_{d}}\times 100\%$$

where $W_{w}$, $W_{t}$ and $W_{d}$ represent the weight of water content, the weight at *t* time and the dry weight of the specimen respectively.

**Figure 2:**
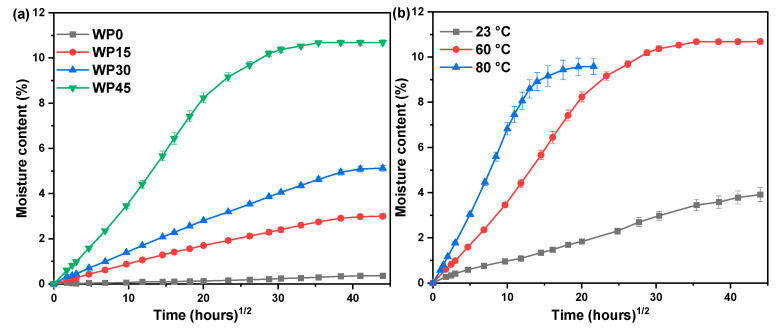
(**a**) Water uptake curves of different samples at a temperature of 60 °C, (**b**) water uptake curves of WP45 at different temperatures.

Figure 2a shows the water content of samples versus the square root of water absorption time at a temperature of 60 °C and every point in the curve represents the average value of five specimens. The pure polypropylene (marked as WP0) is hydrophobic and therefore it absorbs a little water and the moisture content of WP0 can be neglected compared with the composites reinforced with wood powder. The specimens with 15, 30 and 45 wt % wood powder, marked as WP15, WP30 and WP45 respectively, whose water absorption curves show a similar shape. At the first stage of water absorption, the moisture content increased quickly with the immersion time, and the moisture content as a function of the square root of immersion time exhibits an almost linear relationship and then the moisture content increases slowly as the immersion time prolongs until the moisture content of the specimen is saturated. This water absorption behavior is considered to be consistent with Fickian diffusion (Case I) [16,29]. Moreover, it shows that wood powder plays a profound effect on the water absorption behavior of samples, that is, the moisture content increases with the increase of wood powder content, which is also consistent with the studies of other researchers [7,15,17,30]. The moisture content of WP0, WP15, WP30 and W45 is 0.36%, 2.98%, 5.12% and 10.68% respectively when the specimen is in a state of effective moisture equilibrium (allowing the moisture to change within a limited range and a specified period). Furthermore, the equilibrium moisture content as a function of wood content also shows a nearly linear relationship and the correlation coefficient reaches 0.95, which can be confirmed further that wood powder plays a key role in water absorption of the composites. Wood powder, as a kind of lignocellulose materials, can absorb more moisture compared with pure polypropylene due to the existence of the polar group-hydroxyl, therefore, the composites filled with wood powder exhibit better water uptake than pure polypropylene, and the water content increases with wood powder content increasing because the increase of wood powder content means that the existence of a great number of hydroxyl groups. On the other hand, the water absorption behavior of the specimen is also related to the temperature of the immersion environment. The slope of the initial curve indicates that the higher the temperature, the greater the water absorption rate, that is, the temperature can accelerate the water absorption rate of the composite. Moreover, it takes a shorter time to reach the water diffusion equilibrium when in elevated temperatures. The water absorption process is the result of the irregular movement of numerous water molecules. Higher temperatures can accelerate the movement of the molecules and thus reach equilibrium in a short time. As shown in Figure 2b, it took nearly 400 h to get the effective moisture equilibrium at a temperature of 80 °C while the time was about 1200 h at a temperature of 60 °C, and even, the effective moisture equilibrium had not achieved when it took 1600 h at a temperature of 23 °C. Therefore, it can be found that increasing the environmental temperature can accelerate the diffusion rate of water molecules and achieve the effective moisture equilibrium quickly.

As previously noted, the water uptake behavior of the specimen follows Fickian diffusion mechanism and the diffusivity, *D*, can be calculated by the following equation:

(2) $$D=\pi {\left(\frac{h}{4W_{\infty }}\right)}^{2}{\left(\frac{W_{2}-W_{1}}{\sqrt{t_{2}-\sqrt{t_{1}}}}\right)}^{2}$$

where h, $W_{\infty }$, and $\frac{W_{2}-W_{1}}{\sqrt{t_{2}-\sqrt{t_{1}}}}$ represent the thickness of the specimen, the equilibrium moisture content and the slope of the initial section of the moisture absorption curve respectively.

Table 1 shows the diffusion constant of every sample, which also confirms that the water absorption behavior of the specimen is related to wood powder content and temperature, both equilibrium moisture content and the diffusion rate of water molecules increase with the increase of wood powder content, and elevated temperatures can accelerate the diffusion rate of water molecules, thus shortening the time to reach the equilibrium moisture content. Moreover, the specific value of the diffusion constant is consistent with that reported by other researchers, and its order of magnitude remains near 10^−13^ m^2^/s [15,16,31].

From the point of the shape of the curves of the moisture content versus the square root of the immersion time, it can be concluded that the water absorption behavior of the composites can be described by Fickian diffusion (Case I). To further confirm the conclusion, the following equation can be used to distinguish theoretically the diffusion mechanisms:

(3) $$\frac{W_{t}}{W_{\infty }}=kt^{n}$$

where $W_{t}$ represents the moisture content at time *t*, $W_{\infty }$ is the equilibrium moisture content as Equation (2) reports, *k* and *n* are the constants. The various values of *n* represent different moisture diffusion mechanisms as the report in the introduction section. For Fickian diffusion (Case I), *n* = 1/2, for Case II, *n* ≥ 1, and for non-Fickian diffusion (anomalous diffusion), the value of *n* is between 1/2 and 1.

To calculate the value of *n* easily, Equation (3) can be transformed into Equation (4) as follows:

(4) $$log\left(\frac{W_{t}}{W_{\infty }}\right)=\log k+n\log t$$

**Figure 3:**
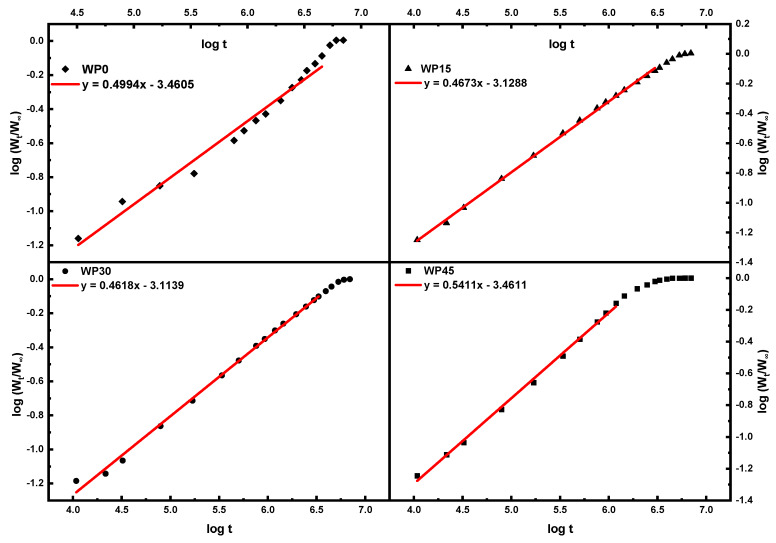
Diffusion case fitting plots for wood-filled polypropylene composites at 60 °C.

According to the equation, *n* represents the slope of the curve of $log\left(\frac{W_{t}}{W_{\infty }}\right)$ versus logt, therefore, it can be obtained by the fitting line of the curve as shown in Figure 3. And the values of *n* of every sample are tabulated in Table 1. The values of *n* for the specimen are close to 1/2, which indicating that the water absorption behavior of wood-filled polypropylene composites follows Fickian diffusion. This can confirm further that the water absorption behavior of natural fiber reinforced polymer composites usually follows Fickian diffusion, as previously reported [15]. As for the difference between the theoretical value and the calculated value, it can be due to other water absorption mechanisms, such as the fiber swelling, the micro gaps in the matrix and the defects in the interface between fibers and matrix [16].

### 3.2. Effect of Water Absorption on the Mechanical Properties of the Composites

**Figure 4:**
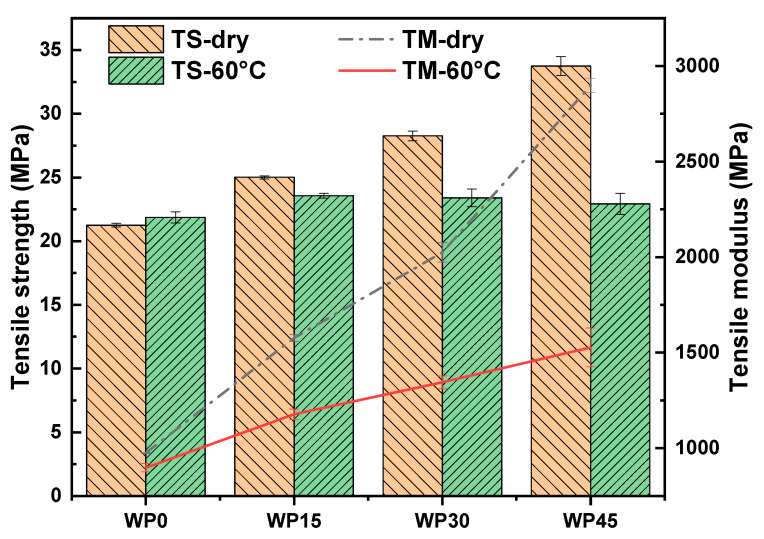
Tensile strength and modulus of samples.

**Figure 5:**
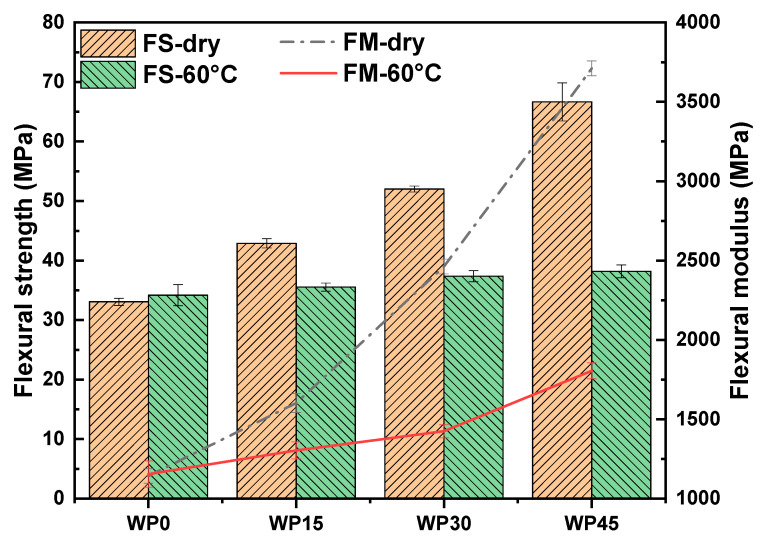
Flexural strength and modulus of samples.

Tensile and flexural tests were performed on samples before and after immersion in distilled water at a temperature of 60 °C. Figure 4 and Figure 5 show the results of tensile and flexural behavior respectively. For pure polypropylene, it is hydrophobic and absorbs very limited water, and the results show that the change of both tensile and flexural behavior can be neglected. However, for wood powder reinforced polypropylene composites, the ability of water absorption is much better than pure polypropylene because wood powder possesses many hydrophilic groups, and the results exhibit that their mechanical properties are degraded. Generally, water absorption can degrade the mechanical properties of the composites. The introduction of water molecules will lead to the change of structure for natural fibers, matrix and the interface between them, such as water-induced fiber swelling, which not only changes the structure of fibers but also causes the interface damaged and the matrix cracked, thus further degrading the mechanical properties of the composites [32]. On the one hand, wood powder swells due to moisture absorption, leading to debonding at the interface, thus affecting stress transfer and the reinforcing effect of wood powder on the matrix. On the other hand, the structure of the polymer matrix is affected due to the penetration of water molecules into polymer chains.

To describe the extent of the hydrothermal aging conditions of the composites, the percentage of the mechanical properties decreased was defined as the aging index (*AI*), therefore, it can be described by the following equation:

(5) $$AI=\left(1-\frac{M_{w}}{M_{d}}\right)\times 100\%$$

where $M_{d}$ and $M_{w}$ represent the values of the mechanical properties of the composites before and after immersion in distilled water respectively. The value of *AI* shows the extent of the aging conditions of the composites, and higher values mean a larger aging extent. Table 2 shows the values of *AI* of the mechanical properties. The symbols *AI*-TS, *AI*-TM, *AI*-FS and *AI*-FM represent the aging index of tensile strength, tensile modulus, flexural strength and flexural modulus respectively. According to the values of *AI*, the hydrothermal aging extent of the composites increases with the increase of wood powder content, and the values of *AI* is almost linearly related to the wood powder content (or water uptake content). The higher wood powder content means more hydrophilic groups and more water absorption percentages. It can be concluded that more water absorption percentages can cause more damage to the mechanical properties of the composites. It is interesting noting that the tensile strength of all composite samples immersed in distilled water is very close to that of pure polypropylene, and the flexural strength of all composite samples shows a similar phenomenon with the tensile strength. This indicates that it is the polypropylene, instead of wood powder, which plays a key role in providing strength after hydrothermal aging of composite samples. Our previous study reported that the flexural strength as a function of wood powder content is nearly linear, which increased with the wood powder content [33]. However, the reinforcing effect of wood powder on polypropylene is little after the samples reach the water absorption balance. This may be due to the damage of wood powder and the destruction of the interface between wood powder and polypropylene, thus affecting the transfer of stress. Compared with strength, the loss of modulus is greater, that is, the stiffness of the composite samples is very sensitive to water, which can not only confirm the damage of water on wood powder and the interface of composites but also prove that water has a plasticizing effect on the polymer matrix [16,23,28,34,35]. However, compared with the modulus of pure polypropylene, the modulus value of wood powder reinforced polypropylene composites is still relatively high. The reason may be that wet wood powder can still play a role in enhancing the matrix’s ability to resist misalignment, especially the lignin in wood powder is hydrophobic, it is not easily degraded by water, and can increase the stiffness of the composite materials.

### 3.3. Dynamic Mechanical Analysis of the Composites

Dynamic mechanical analysis (DMA) is a common method to characterize the viscoelastic properties of polymer materials, besides, it can be used to exhibit the cure behavior and the molecular mobility of polymer materials [36]. In this study, DMA is used to study the characteristics of the interface between wood powder and polypropylene with the following parameters: the storage modulus, the loss factor and the glass transition temperature (*T*_g_). It is noted that the glass transition temperature can be determined by the peak temperature of the loss factor curve.

**Figure 6:**
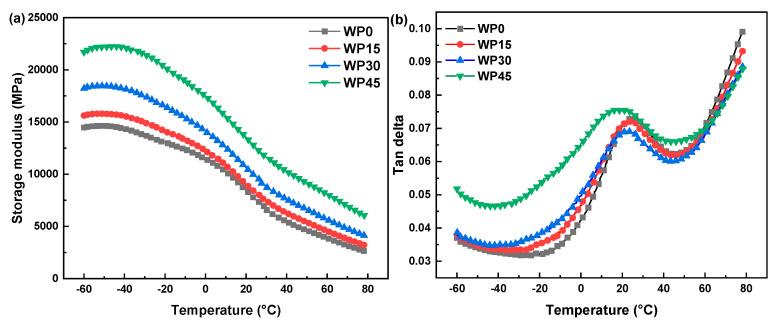
Dynamic mechanical analysis (DMA) test curves of the samples immersed in distilled water at a temperature of 60 °C: (**a**) storage modulus and (**b**) tan delta.

The storage modulus and loss factor (tan delta) of the specimens immersed in distilled water at a temperature of 60 °C are presented in Figure 6. As shown in Figure 6a, although the value of each sample is different, all four curves show similar behavior, and the rank of the values of the storage modulus for these four samples is WP45, WP30, WP15 and WP0 in sequence, which is in agreement with Young’s modulus and the flexural modulus discussed in the previous section. It proves again that wood powder after hygroscopicity still has a positive effect on the stiffness of the composites. As mentioned above, the lignin in wood powder is hydrophobic and rigid, and water molecules have little effect on it. Moreover, the value of storage modulus decreases with the increase of the test temperatures, which indicates that the stiffness of the samples reduces when the materials transform from the glassy state into the rubbery state. This is because frozen polymer segments gain energy at higher temperatures, and begin to thaw and move. At the same time, the mobility of the polymer segments makes the viscosity of the materials increased, and the higher tan delta indicates the higher viscoelastic damping character, which can be found in Figure 6b. It is worthy to note that the values of tan delta for WP15 and WP30 are very close to that of pure polypropylene in the initial glassy state, which is much less than the value of tan delta for WP45. This shows that the bonding of the interface for WP15 and WP30 is better than that of the interface for WP45, which can also be confirmed by comparing the peaks of the loss factor curves. More wood powder easily leads to the agglomeration of particles, which is not conducive to the dispersion of wood powder in the matrix, thereby reducing the adhesion of the interface between the wood powder and the matrix. According to the peak temperature of the loss factor shown in Figure 6b, the glass transition temperature of all samples can be determined. It can be found that the glass transition temperature of sample WP0 (pure polypropylene) is the highest, and the next is WP15 and WP30, but they are close to WP0, and WP45 is the lowest. This indicates that the incorporation of wood powder decreases the glass transition temperature of the composite samples, and such a similar result that the glass temperature of composites is lower than that of pure resin is also found by other studies [36,37,38].

**Figure 7:**
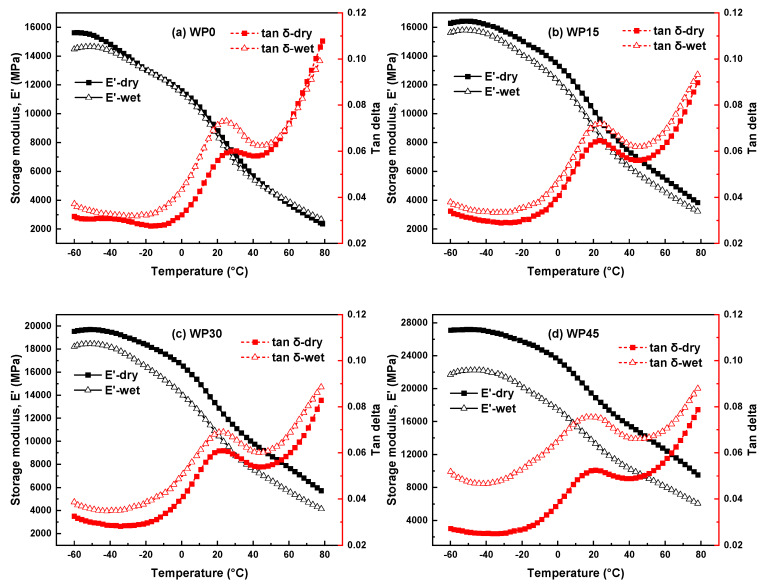
The storage modulus and loss factor of the specimens immersed in distilled water and corresponding dry specimens: (**a**) WP0, (**b**) WP15, (**c**) WP30 and (**d**) WP45.

Figure 7 shows the comparison for the storage modulus and loss factor of the specimens immersed in distilled water at a temperature of 60 °C and corresponding dry specimens. As shown in Figure 7a, except for the difference at the beginning of the glassy state, the storage modulus curve of the dry specimen is almost superposed to that of the wet sample, which can confirm that the water molecules have little effect on the storage modulus of polypropylene. This is consistent with the results of tensile and bending tests. As for the difference at the initial of the glassy state, it can be explained that the penetration of water molecules made the distance of polymer chains enlarge, thus increasing the free degree of stiffness groups and lowing the stiffness of the material [35]. For other wood powder reinforced polypropylene composites (WP15, WP30 and WP45), the storage moduli of the dry specimens are notably higher than those of the wet samples regardless of in the glassy state, the glass-rubber transition state or the rubbery state, and the difference of the storage modulus between dry and wet samples becomes larger with the increase of wood powder content. More specifically, the storage modulus in the glassy state decreased by 3.85%, 6.32%, 18.21% for WP15, WP30 and WP45 respectively. Besides, the storage modulus in the rubbery state exhibited a similar difference between dry and wet samples with that in the glass state. This is consistent with the results obtained by the tensile and flexural tests, that is, the more water the sample has, the greater the loss of its mechanical properties. On the other hand, in respect of the loss factor (tan delta), the value of the loss factor of the wet sample is higher than that of the corresponding dry sample for all specimens. Especially for WP45, the difference of tan delta for dry and wet samples is the highest. The loss factor presents the viscoelastic damping character of the material, and it can characterize the adhesion of the interface between wood powder and polypropylene to some extent. So, a higher value of the loss factor indicates the higher damping and poor adhesion of the interface, that is, the introduction of water molecules makes the elastic properties of the samples decrease and makes the viscidity increase. More importantly, the water molecules have an adverse effect on the interface, as well as shifting the glass transition temperature towards the lower temperature slightly. Although the values of tan delta for the wet polypropylene and the wet composites are higher than that of tan delta for corresponding dry samples, the mechanisms of damage are diverse. For pure polypropylene, as discussed earlier, water molecules play the role of plasticizers. The entry of water molecules increases the degree of freedom of the polymer segment, thereby increasing the viscosity of the material. As for the wood powder reinforced polypropylene composites, the water molecules may cause the crack and debonding in the interface area due to the mismatch of the moisture expansion coefficients between wood powder and polypropylene, which would cause higher energy dissipation [36,39].

## 4. Conclusions

This paper studied the water absorption behavior of wood powder reinforced polypropylene composites and the effect of hydrothermal aging treatment on the mechanical properties of composites. It was found that the moisture absorption process of wood powder reinforced polypropylene composites complied with Fick’s law, and the diffusion coefficient of water had a positive correlation with the ambient temperature and the wood powder content in the material. It took a shorter time to achieve the effective moisture equilibrium at the elevated temperatures for the same specimen. Similarly, at the same ambient temperature, samples with more wood powder content can quickly achieve moisture absorption balance. Although the hygroscopic process followed Fick diffusion and Fick diffusion played a major role in the water absorption of the composite, the hygroscopic process of the material was the result of the combined action of multiple hygroscopic mechanisms. This was because the results calculated from the experimental data were slightly different from the theoretical values different. The tensile and flexural strength of wood powder reinforced polypropylene composites with saturated moisture content mainly depended on the polypropylene matrix and wood powder had little reinforcing effect on the matrix. However, the wood powder can still play a role in increasing the stiffness of the composite material, which can be seen from the values of the tensile modulus and flexural modulus that were much higher than those of pure polypropylene. This was also confirmed by the dynamic mechanical analysis of the material. More specifically, the storage modulus increased with the increase of the wood powder content. In addition, the analysis of dynamic mechanical properties showed that after the water absorption, the glass transition temperature of the composite samples decreased, indicating that the thermal stability decreased.

## Figures and Tables

**Table 1:** Water absorption parameters for wood-filled polypropylene composites.

Samples	Diffusion Constant (m^2^/s)	*n ^a^*
WP0-60 °C	1.90 × 10^−13^	0.4993
WP15-60 °C	3.41 × 10^−13^	0.4673
WP30-60 °C	3.20 × 10^−13^	0.4618
WP45-60 °C	7.35 × 10^−13^	0.5411
WP45-80 °C	24.92 × 10^−13^	0.5394

^a^ n is the exponent in Equation (3) and is related to the mode of diffusion.

**Table 2:** The values of *AI* for different mechanical properties.

Samples	*AI*-TS (%)	*AI*-TM (%)	*AI*-FS (%)	*AI*-FM (%)
WP15	5.79	25.31	17.18	18.63
WP30	17.20	33.60	28.18	42.14
WP45	32.06	47.30	42.72	51.35
